# MicroRNAs as Biomarkers for Adenomyosis: A Systematic Review

**DOI:** 10.3390/biomedicines14081764

**Published:** 2026-08-05

**Authors:** Paula Buehler, Angela Vidal, Cloé Vaineau, Tanya Karrer, Michael Mueller

**Affiliations:** 1 Department of Gynecology and Obstetrics, Bern University Hospital, University of Bern, 3010 Bern, Switzerland; 2 Division of Gynecological Endocrinology and Reproductive Medicine, Bern University Hospital, University of Bern, 3010 Bern, Switzerland; 3 Medical Library, University Library of Bern, University of Bern, 3012 Bern, Switzerland

**Keywords:** adenomyosis, microRNA, biomarker

## Abstract

**Background/Objectives**: Adenomyosis is a chronic gynecological disorder characterized by the presence of endometrial tissue within the myometrium, causing pelvic pain, abnormal uterine bleeding, and infertility. Despite its high prevalence, the molecular mechanisms underlying disease initiation and progression remain incompletely understood. Current evidence implicates disruption of the endometrial–myometrial interface, epithelial–mesenchymal transition, and progesterone resistance in driving tissue invasion and remodeling. Diagnosis relies mainly on imaging modalities, while reliable non-invasive biomarkers are lacking. MicroRNAs, as stable post-transcriptional regulators of gene expression, have emerged as key modulators of proliferation, inflammation, and hormonal signaling, and represent promising candidates for novel diagnostic strategies. **Methods**: A systematic review was conducted in accordance with PRISMA guidelines and registered with PROSPERO (CRD42025637752). A comprehensive search of the Medline, Embase, Scopus, and Cochrane databases was performed in April 2025. Studies investigating miRNA expression in patients with adenomyosis compared with controls were included. The quality of the studies and the risk of bias were assessed using the Newcastle–Ottawa scale. Two reviewers independently performed study selection, data extraction, and quality assessment. **Results:** Twenty-seven studies published between 2015 and 2025 met the inclusion criteria. Thirty-nine distinct miRNAs were reported as significantly dysregulated in adenomyosis. Recurrently altered miRNAs included let-7a, miR-145, miR-10b, miR-30c-5p, miR-141-3p, miR-143, and miR-191. Functional analyses have consistently implicated miRNAs in key pathogenic pathways, including Hippo-YAP, PI3K/AKT, MAPK/ERK, JAK/STAT, and Wnt/β-catenin signaling. These alterations were associated with enhanced epithelial–mesenchymal transition, increased cellular proliferation and migration, progesterone resistance, chronic inflammation, and immune modulation. Emerging evidence highlights exosomal and circulating miRNAs as promising non-invasive biomarkers, with a few studies already demonstrating diagnostic potential using serum, plasma, or urine samples. However, substantial heterogeneity in tissue types, sampling timing, and analytical methods precluded meta-analysis. **Conclusions**: MiRNAs play a central role in the molecular pathogenesis of adenomyosis and show strong potential as non-invasive diagnostic biomarkers. However, large-scale validation studies and standardized methodologies are required before clinical implementation.

## 1. Introduction

Adenomyosis (AM) is a benign, chronic gynecological disorder defined by the ectopic localization of endometrial glands and stroma within the myometrium of the uterus. This pathology primarily affects women of reproductive age and is frequently associated with clinical manifestations including dysmenorrhea, abnormal uterine bleeding, and infertility [1]. Epidemiological investigations have reported a prevalence of 5–70% in hysterectomy specimens, with the most commonly cited range being 20–35%. Of note, an ultrasound-based investigation by Naftalin et al. reported a prevalence rate of 20% [1,2,3].

Despite its considerable impact on reproductive health and quality of life, the etiology and pathogenesis of AM remain incompletely elucidated [4,5]. Current evidence suggests that the pathogenesis of AM is multifactorial, involving several interrelated biological mechanisms. The pathological process predominantly affects the inner myometrium, also referred to as the junctional zone (JZ), which constitutes a hormonally responsive region responsible for modulating uterine peristalsis. Two principal pathogenetic pathways have been described. The prevailing hypothesis posits that invagination of the basal endometrium into the myometrium is facilitated by repetitive tissue injury and repair (TIAR), uterine hyperperistalsis, and progressive compromise of the endometrial–myometrial interface (EMI). Epithelial–mesenchymal transition (EMT) further potentiates this process by enabling endometrial epithelial cells to lose intercellular adhesion, acquire migratory properties, and assume mesenchymal phenotypes. Alternatively, adenomyotic lesions may develop de novo through the metaplastic transformation of embryonic Müllerian remnants within the myometrium or via aberrant migration and differentiation of endometrial stem or progenitor cells. Genetic and epigenetic modifications, such as progesterone receptor silencing and dysregulated estrogen receptor signaling, are also thought to contribute to disease onset, progression, and persistence [4,6,7].

Diagnosis of AM relies on a combination of gynecological examination, imaging modalities, pathological assessment, and, in some cases, hysteroscopy. Currently, transvaginal ultrasound (TVUS) and magnetic resonance imaging (MRI) represent the most informative imaging techniques. While MRI demonstrates higher sensitivity (83–92%) compared to TVUS (74–81%), it remains a costly and time-intensive procedure [8]. Consequently, there is growing interest in identifying biomarkers as supplementary diagnostic tools for AM. In AM, biomarker research has so far been unsuccessful, as the existing candidates are nonspecific and cannot reliably differentiate the disease [1].

MicroRNAs (miRNAs) are a class of non-coding RNAs, comprising small, single-stranded molecules approximately 20–24 nucleotides in length, that regulate gene expression at the post-transcriptional level by binding to target mRNAs and modulating their splicing, degradation, and translation [9]. Notably, a single miRNA can influence the expression of several hundred genes. MiRNAs are involved in a wide array of biological processes, including cell division, proliferation, differentiation, apoptosis, and responses to cellular stress. Due to their remarkable stability in biological fluids and tissue-specific expression patterns, miRNAs are considered promising biomarker candidates [9,10,11]. Their utility as biomarkers has already been demonstrated in related gynecological disorders, such as endometriosis (e.g., EndoTest^®,^ Ziwig, Lyon, France), as well as in oncology and various other medical fields [12,13,14,15,16].

The objective of this review is to systematically summarize and critically evaluate the current knowledge regarding the role of miRNAs in the pathogenesis of AM, as well as their potential utility as diagnostic and therapeutic biomarkers.

## 2. Materials and Methods

### 2.1. Registration of Protocols

The protocol was registered with the Prospective International Registry of Systematic Reviews (PROSPERO; registration number CRD42025637752). The Preferred Reporting Items for Systematic Reviews and Meta-Analyses (PRISMA) guidelines were used (Table S3) [17].

### 2.2. Eligibility Criteria, Information Sources and Search Strategy

**Figure 1:**
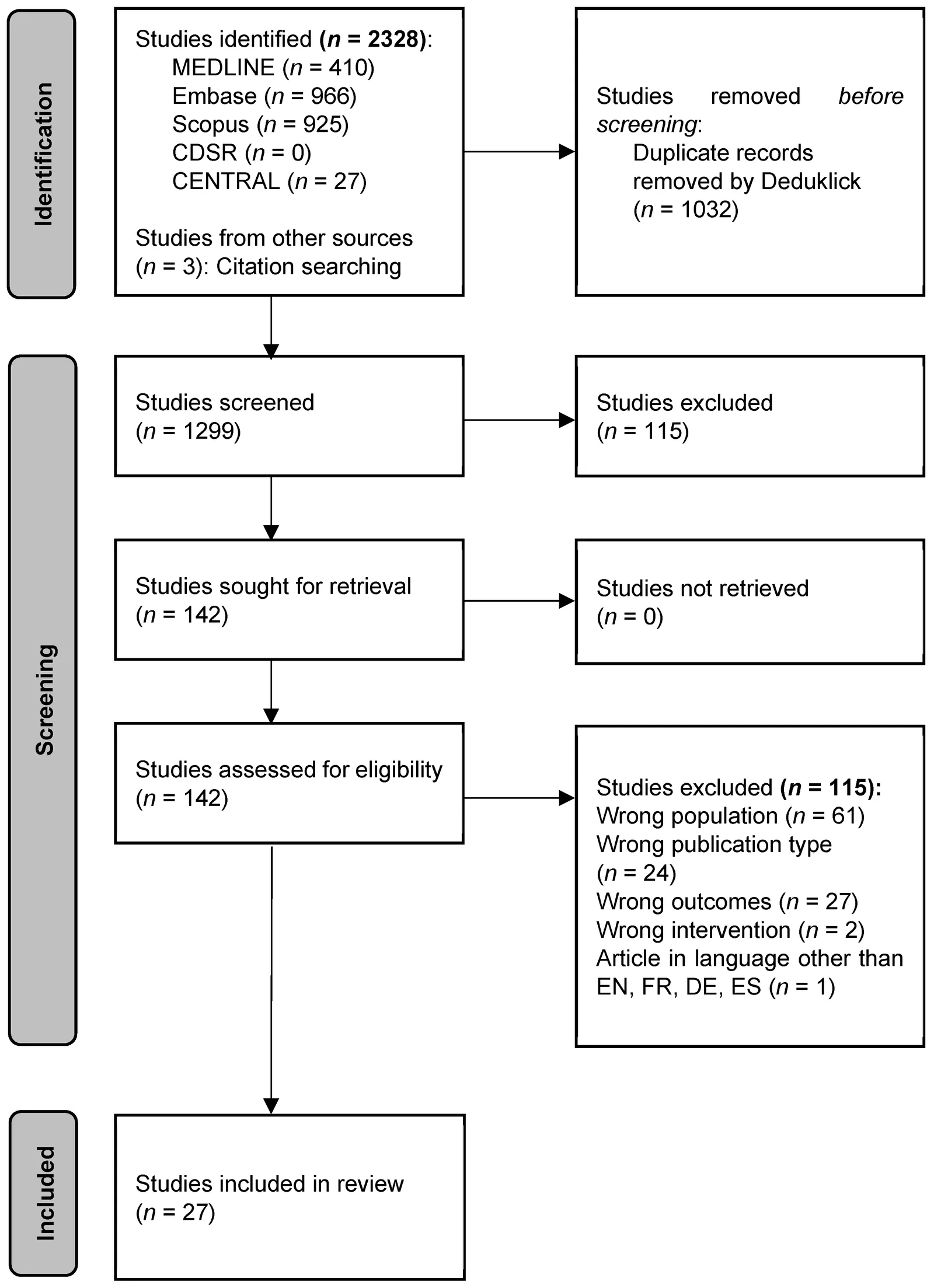
PRISMA flow chart of evaluated studies.

To identify potentially relevant publications on the topic, a search strategy was developed and conducted in MEDLINE, Embase, Scopus, and the Cochrane Database of Systematic Reviews and Central Register of Controlled Trials (CENTRAL). A medical information specialist developed an initial search strategy in Embase for the concepts microRNA and adenomyosis. We tested the search strategy against a list of core references to ensure that key publications were included. After refinement, the information specialist set up the search strategy for each information source based on database-specific index terms and free text. The free-text search included synonyms, acronyms, and similar terms. In Medline and Embase, a “humans only” filter was applied using a double-negative search strategy suggested by the Ovid database guides for Medline and Embase, respectively. We excluded animal studies from the Scopus search with an adapted version of the Ovid double-negative filter. Publication years were limited to 2000 to the present, and languages to English, French, German, or Spanish. No other database-provided limits were applied across sources, including study types or other formal criteria. The search was performed on 29 April 2025. Results were deduplicated using Deduklick (https://www.risklick.ch/deduklick, accessed on 29 April 2025) [18] and imported into Covidence (https://covidence.org, accessed on 29 April 2025) for screening. The study selection process was depicted using the PRISMA flowchart, as shown in Figure 1. The complete database-specific search strategies for all databases are provided in Supplementary Material File S1.

### 2.3. Selection Process

Studies were independently assessed for inclusion using Covidence software (www.covidence.org, accessed on 29 April 2025) [19] by the investigators PB and AV. Eligibility was determined based on original publications reporting miRNAs as biomarkers in the context of AM. Only studies that examined miRNAs and AM were considered. Studies were included only if they used a case–control design to investigate differences in microRNA expression between patients with AM and controls. We excluded reviews, conference abstracts, case reports, abstracts without full-text data, and studies unrelated to AM or without primary microRNA data. Publications not available in English were also excluded from the analysis. All eligibility criteria were specified in advance of the study selection process.

### 2.4. Data Collection Process and Data Items

The extracted data were independently summarized by two investigators (PB and AV) and subjected to a detailed review. The primary variables included characteristics of the study populations such as patient age, phase of the menstrual cycle, prior hormone therapy, and method of diagnosis. Other variables included sample collection and statistical analysis. Disagreements were discussed and resolved by consensus.

### 2.5. Study Risk-of-Bias Assessment

The Newcastle–Ottawa Scale (NOS) was used to assess the quality of the individual studies [20]. Three parameters were considered for individual study scoring: subject selection (0–4 stars), comparability (0–2 stars), and study outcome (0–3 stars). The scoring was composed as follows: good quality (3 or 4 stars in the selection domain AND 1 or 2 stars in the comparability domain AND 2 or 3 stars in the outcome/exposure domain), fair quality (2 stars in the selection domain AND 1 or 2 stars in the comparability domain AND 2 or 3 stars in the outcome/exposure domain), and poor quality (0 or 1 star in the selection domain OR 0 stars in the comparability domain OR 0 or 1 star in the outcome/exposure domain). All included studies were independently reviewed by PZ and AV to assess the risk of bias. Disagreements were resolved by consensus.

### 2.6. Effect Measures, Synthesis Methods and Certainty Assessment

Due to the considerable heterogeneity of the study populations, methods, outcome definitions, and reported performance measures, a meta-analysis was not feasible. Therefore, a narrative synthesis was performed. The included studies were grouped according to the outcomes investigated and relevant study characteristics. Diagnostic performance measures, including sensitivity, specificity, area under the ROC curve (AUC), and odds ratios (ORs), were extracted and descriptively compared. The results were summarized both in text and in tables to highlight similarities and differences between the studies.

A formal assessment of the certainty of evidence was not undertaken, given the heterogeneity of the included studies and the absence of a meta-analytic synthesis. As a result, the overall certainty of the evidence for each outcome was not formally established.

## 3. Results

### 3.1. Study Selection, Study Characteristics

This review aims to elucidate the role of miRNAs in the pathogenesis of AM, with particular emphasis on their potential as diagnostic biomarkers. Accordingly, our inclusion criteria were limited to studies that examined miRNA expression profiles in various biological tissues obtained from individuals with AM and control subjects. A total of 27 studies that met these criteria were included in our review. All these studies were published within the last decade, specifically between 2015 and 2025. Table 1 lists the miRNAs that showed statistically significant differences between AM patients and the control group. MiRNAs cited multiple times throughout the literature are emphasized in bold.

Most of the evaluated studies are observational and preclinical. They primarily investigated the role of miRNA in the pathophysiology of AM. Six studies also explored the potential use of miRNA as a new diagnostic tool. Some studies have established a correlation between upregulated or downregulated miRNAs and symptoms, and even symptom severity. Overall, significant differences between AM patients and controls were found for 39 miRNAs (listed in Table 2), which were investigated either in a pathophysiological context to elucidate disease mechanisms or in a diagnostic context to evaluate their potential as biomarkers. Table 2 also highlights miRNAs that were investigated across different specimen types, reported in multiple studies, or showed inconsistent expression patterns. Notably, miR-124-3p and miR-145 were assessed in multiple studies and exhibited conflicting expression patterns in endometrial tissue, with some studies reporting upregulation and others downregulation.

The included studies demonstrated considerable methodological heterogeneity. Most investigations focused on miRNA expression in endometrial tissue, although sample collection methods varied, including hysterectomy, endometrial curettage, and biopsy. Additional studies examined serum, plasma, or urine samples. Moreover, some research focused on whole tissue, while others analyzed exosomes or isolated cell populations. An additional potential bias arises from differences in diagnostic criteria for AM and in the definitions of case and control groups across the studies. Further information is provided in Supplementary Table S1. The reporting of menstrual cycle phase during sample collection was inconsistent, with substantial variation across studies.

### 3.2. The Role of MicroRNAs in Key Signaling Pathways Involved in the Pathogenesis of Adenomyosis

Across various studies, the fundamental signaling pathways involved in the pathogenesis of AM are progressively being elucidated. This pathogenesis is marked by a persistent dysregulation of specific miRNAs that target key signaling pathways governing proliferation, epithelial–mesenchymal transition (EMT), inflammation, and hormonal signaling.

A central finding is the loss of tumor-suppressive miRNAs, which leads to activation of the Hippo-YAP, PI3K/AKT, MAPK/ERK, JAK2/STAT3, and Wnt/β-catenin pathways. These alterations promote proliferation, migration, invasion, and resistance to apoptosis, supporting the concept of AM as an actively remodeling disease rather than a passive structural displacement. EMT consistently emerges as a key mechanism enabling myometrial invasion.

The Lin28B/Let-7a axis is a central regulator of proliferation in junctional zone smooth muscle cells (JZSMCs). Let-7a normally inhibits cell growth, whereas Lin28B overexpression suppresses Let-7a and enhances proliferation. Lin28B knockdown restores Let-7a levels and reduces JZSMC growth. Reduced Let-7a activates the Hippo-YAP1 pathway by increasing YAP1 and TAZ expression, thereby promoting proliferation and inhibiting apoptosis. This contributes to smooth muscle hyperplasia and uterine enlargement. Furthermore, the data suggest that 17β-estradiol interacts with the Lin28B/Let-7a axis, potentially amplifying proliferative signaling and contributing to AM development [27,29,30].

Furthermore, miR-141 functions as an inhibitor of the Lin28B/let-7a axis. The circular RNA circ_0061140 is significantly upregulated in adenomyotic tissues, where it acts as a molecular sponge, suppressing the expression of miR-141-3p. Decreased levels of miR-141-3p increase LIN28B expression. As previously demonstrated by Li et al., elevated LIN28B inhibits the expression of let-7, thereby augmenting proliferative signaling and contributing to disease progression [35].

Numerous studies establish a correlation between miRNA dysregulation and sustained activation of the phosphoinositide 3-kinase (PI3K)/protein kinase B (AKT) pathway. The downregulation of miR-10b results in increased expression of PIK3CA and enhanced phosphorylation of AKT, thereby augmenting cellular migration, invasion, and epithelial–mesenchymal transition (EMT) through ZEB1-mediated repression of E-cadherin. Conversely, elevated levels of miR-17 activate the pathway by targeting Phosphatase and tensin homolog (PTEN), thereby eliminating its inhibitory effect and fostering proliferation and cell survival [21,23]. Extracellular vesicle-associated miR-25-3p and miR-92a-3p further potentiate AKT signaling by suppressing PTEN, thus facilitating macrophage M2 polarization, angiogenesis, cellular migration, and EMT [40,44]. In summary, these miRNA alterations collectively contribute to the persistent activation of the PI3K/AKT pathway.

The MAPK/ERK pathway is similarly regulated by opposing miRNAs. miR-4669 enhances ERK signaling via DUSP6 suppression, whereas miR-30c-5p inhibits MAPK1. Their imbalance sustains ERK activation, macrophage polarization, EMT, and invasiveness [34,43].

Aberrant JAK2/STAT3 signaling is driven by downregulation of tumor-suppressive non-coding RNAs, including MIR22HG, miR-2861, and miR-141-3p. This increases STAT3 expression or phosphorylation, resulting in enhanced proliferation and reduced apoptosis of endometrial and smooth muscle cells [33,41].

Finally, activation of the Wnt/β-catenin pathway is a major driver of EMT and invasiveness. Downregulation of miR-145-5p, together with upregulation of miR-191, sustains β-catenin activity and mesenchymal transformation. Dysregulation of the MIR503HG/miR-191 and miR-145-5p/Talin1 axes therefore contributes to pathological uterine remodeling via Wnt/β-catenin-mediated EMT [32,38].

### 3.3. MicroRNAs as a Diagnostic Approach in Adenomyosis

Six of our twenty-seven studies analyzed the potential of miRNAs as diagnostic biomarkers for AM in endometrial tissue, peripheral blood, and extracellular vesicles in serum, plasma, and urine.

First, in 2020, Borisov et al. [25] identified miR-10b, miR-200c, and miR-191 as significantly dysregulated in the eutopic endometrium. By calculating expression ratios of reciprocally regulated miRNAs, diagnostic sensitivities of 61–74% and specificities of 72–86% were achieved, suggesting that endometrial miRNA profiling may represent a new diagnostic approach across studies.

In addition, dysregulation of the circRNA–miRNA–mRNA network involving miR-124-3p was described. Increased miR-124-3p expression, together with reduced circ_0008959 and SLC15A4 levels in eutopic endometrial tissue, was associated with AM, and combining molecular markers with clinical parameters improved diagnostic performance. In Guo et al., the highest diagnostic accuracy was achieved by the combined model (circ_0008959, SLC15A4, and miR-124-3p) including the VAS score (sensitivity: 91%; specificity: 97%), while miR-124-3p demonstrated the best performance among the single biomarkers [39].

Circulating miRNAs show even greater diagnostic promise. A recent pilot study by Kupec et al. investigated serum and urine miRNA profiles for the non-invasive diagnosis of AM using next-generation sequencing combined with machine learning. Distinct biofluid-specific miRNA signatures were identified, with miR-183-3p, miR-17, and miR-320d-2 emerging as promising candidate biomarkers, particularly in urine samples, which showed superior discriminatory performance [46]. In the study by Shao et al., miR-92a-3p was significantly elevated in AM and in plasma exosomes, ectopic lesion exosomes, and urinary exosomes, with the highest diagnostic accuracy achieved for urinary exosomes. The concentrations correlated with clinical severity, thus supporting the suitability of miR-92a-3p as a non-invasive biomarker for disease detection and monitoring [44].

The strongest diagnostic performance was observed with a serum-based 2-miRNA panel comprising miR-101-3p and miR-143-3p. Individually, these miRNAs showed AUC values of 0.881 and 0.901, respectively. When combined, the panel reached an AUC of 0.941, with a sensitivity of 93.33% and specificity of 96.67%. The panel also demonstrated strong discrimination between AM and other gynecological conditions, and serum miRNAs remained stable under different storage conditions [45].

In Table 2, we provide an overview of the miRNAs significantly dysregulated in different tissue and fluid samples from patients with AM, as analyzed in our studies. In this table, we highlight that some miRNAs have been identified in multiple studies and across different tissue types, underscoring their potential as diagnostic biomarkers. Although it is not yet possible to deduce which miRNA combinations might offer the greatest diagnostic potential across the different studies, this table helps synthesize these findings.

Overall, endometrial miRNA signatures provide moderate diagnostic accuracy, while circulating and exosomal miRNAs—particularly serum-based panels—show higher diagnostic performance and represent promising non-invasive biomarkers for AM.

### 3.4. Risk of Bias in Studies

The quality of the studies was assessed using the Newcastle–Ottawa Quality Assessment Scale for Case–Control Studies. The case–control aspect was evaluated for all studies. Overall, the methodological quality of the included studies was fair. Most studies demonstrated adequate case definitions and objective laboratory-based exposure assessment. However, comparability between cases and controls was often limited by the absence of adjustment for multivariable confounders and reliance on hospital-based control groups. None of the studies reported non-response rates. Therefore, while mechanistic findings are robust, clinical epidemiological strength remains moderate. The detailed results of the quality assessment are provided in Supplementary Table S2.

## 4. Discussion

The primary aim of this study was to investigate the potential correlation between AM and differential miRNA expression to identify molecular signatures involved in its pathogenesis, clinical manifestations, and diagnostic potential.

Our review showed consistent miRNA alterations in both eutopic and ectopic endometrial tissues, suggesting that these small non-coding RNAs play a central role in the aberrant cellular processes that characterize AM, such as uncontrolled proliferation, enhanced migratory capacity, and chronic inflammation [11,21,23,26,31,32,33,34,35,36,38,41,42,44]. In turn, dysregulated miRNAs modulate key signaling pathways, including Hippo-YAP, PI3K/AKT, MAPK/ERK, JAK2/STAT3, and Wnt/β-catenin, indicating that AM is characterized by active miRNA-driven tissue remodeling rather than merely a structural uterine disorder [47].

Given their stability in biofluids and tissue specificity, miRNAs are promising candidates for developing non-invasive diagnostic biomarkers and potential therapeutic targets in AM.

Importantly, several of the dysregulated miRNAs in AM have also been extensively reported in endometriosis, reinforcing the concept of a shared molecular framework between the two estrogen-dependent disorders [48]. It is estimated that up to 80% of patients with AM also present with endometriosis, and their coexistence is clinically associated with increased symptom burden, more severe dysmenorrhea, chronic pelvic pain, and treatment resistance [49,50]. From a molecular perspective, miRNAs such as miR-21, let-7, and miR-145 are commonly altered in both diseases and modulate overlapping biological processes, including immune activation, extracellular matrix remodeling, and fibrosis [27,29,30,32].

Infertility is a significant clinical consequence of AM, often linked to impaired endometrial receptivity, altered uterine contractility, and chronic inflammation. Evidence points to progesterone resistance as a key mechanism that disrupts decidualization and embryo implantation, as reported by Yan et al. These findings highlight miRNA-mediated progesterone resistance as a molecular link between AM and infertility and suggest novel diagnostic and therapeutic targets [24,51].

Over the past decade, circulating miRNAs have attracted considerable interest as minimally invasive biomarkers owing to their molecular stability and disease-specific expression patterns. In cardiovascular research, their diagnostic and prognostic potential was recognized more than a decade ago, including as predictors of response to cardiac resynchronization therapy. However, despite encouraging findings, miRNA-based biomarkers have not yet entered routine clinical practice for heart failure, illustrating the challenges of translating promising biomarker discoveries into clinically validated diagnostic tools [15,16].

In endometriosis, translational progress has already been achieved through the development of clinically validated assays such as EndoTest^®^, which utilizes salivary miRNA signatures for diagnosis [12,52,53]. By contrast, several promising circulating miRNAs have been identified in serum and urine for AM [44,45,46]. However, before a comparable diagnostic test can be established, large multicenter validation studies using standardized diagnostic criteria are required. In addition, reproducible miRNA signatures with predefined cut-offs, external validation, clear discrimination from related gynecological disorders, and demonstrated clinical utility will be essential for successful clinical implementation.

Future research should focus on translating exploratory miRNA profiling into functional validation and clinical application. Although numerous miRNAs have been implicated in epithelial–mesenchymal transition, inflammation, progesterone resistance, and impaired decidualization, their precise mechanistic roles remain incompletely understood. Accordingly, advanced experimental models, including organoids, single-cell transcriptomics, and in vivo systems, will be essential for elucidating miRNA–target networks and cell-type-specific functions within the uterine microenvironment.

Large prospective multicenter studies are required to validate candidate miRNAs as reliable minimally invasive biomarkers using liquid biopsy platforms such as plasma, urine, or saliva. Integrating miRNA expression profiles with clinical and imaging data may improve diagnosis, disease stratification, and personalized patient management. Machine learning (ML) techniques could help identify complex multidimensional biomarker signatures from high-dimensional sequencing datasets and improve diagnostic and prognostic prediction models. However, successful clinical translation will require standardized detection platforms, harmonized analytical workflows, and validation in large, independent cohorts before routine clinical implementation.

Furthermore, investigating the relationship between miRNA dysregulation and reproductive outcomes—including implantation failure, infertility, and assisted reproductive technology (ART) success—may identify predictive biomarkers and support fertility-preserving strategies. The identification of therapeutically actionable miRNAs also offers considerable potential for RNA-based therapies targeting dysregulated molecular pathways in AM. Supporting this translational direction, the ongoing ADENO-MIRNA clinical trial (ClinicalTrials.gov Identifier: NCT06373822) aims to characterize circulating miRNA signatures in patients with AM to establish novel diagnostic biomarkers.

Our study presents several limitations. First, many of the included studies were based on small, single-center cohorts. Second, there was substantial heterogeneity in study design, including variability in sample types, normalization methods, miRNA detection platforms, and cycle-phase matching, which complicates direct comparison and synthesis of findings. Moreover, many studies lacked functional validation. Taken together, these factors meant that meta-analysis was not feasible, and the conclusions drawn must be interpreted with caution.

In conclusion, our findings underscore the critical role of miRNAs in the pathophysiology of AM, both in local tissue remodeling and systemic manifestations such as infertility. Their overlap with endometriosis highlights their diagnostic and therapeutic potential. The detection of these miRNAs in accessible biofluids opens the door to non-invasive, cost-effective diagnostic approaches and supports personalized medicine in gynaecologic disorders.

## Figures and Tables

**Table 1:** Differentially regulated microRNAs (miRNAs) in adenomyosis (AM), their roles in pathology, their diagnostics and their clinical relevance.

Author, Year	Sample Type and Phase of the Menstrual Cycle if Mentioned	Method of MicroRNA Detection	Number of Tested MicroRNAs	Upregulated MicroRNAs (↑)	Downregulated MicroRNAs (↓)	Main Findings Regarding MicroRNA and Adenomyosis Pathology	Main Findings Regarding MicroRNA and Adenomyosis Diagnostics, Clinical Evaluation
Guo, 2015 [21]	Eutopic and ectopic endometrial tissue (sample taken after hysterectomy), different menstrual phases	Screening: microarray; validation: RT-qPCR	Screening: 1387; validation: 10	miR-143 miR-466 miR-513a	miR-10b miR-30c miR-371b-5p miR-92b-5p	miR-10b↓ → ZEB1↑ and PIK3CA↑ → E-Cadherin↓/p-Akt↑ → migration, invasion↑	Not investigated
Herndon, 2016 [22]	Eutopic endometrial tissue (sample taken after hysterectomy), proliferative phase	Microarray	Not specified	miR-9-1 miR-139 miR-149 miR-197 miR-326 miR-339	None	Not specified	Not investigated
Hu, 2017 [23]	Eutopic endometrial tissue (surgical biopsy)	RT-qPCR	1	miR-17	None	miR-17↑ → PTEN↓ → invasion↑, apoptosis↓	Not investigated
Yan, 2019 [24]	Eutopic endometrial tissue (biopsy), secretory phase (cycle day 19–23)	RT-qPCR	1	None	miR-21	miR-21↓ → ↑KLF12, ↓NR4A1 → impaired decidualization, risk of infertility↑	Not investigated
Borisov, 2020 [25]	Eutopic endometrial tissue (pipelle), proliferative phase (cycle day 6–13)	Screening: universal RT-qPCR; validation: ttRT-qPCR (two-tailed primers)	Screening: 170; validation: 9	miR-191	miR-10b miR-200c	Not investigated	Ratios of reciprocally regulated miRNAs show higher diagnostic potential than single miRNAs. The miR-181b/miR-10b expression ratio effectively differentiates AM patients from healthy controls with high diagnostic accuracy (AUC = 0.77; sensitivity = 61.29%; specificity = 72.41%).
Liang, 2020 [26]	Eutopic endometrial tissue (endometrium sample)	RT-qPCR	1	miR-17	None	lncRNA H19↓ → miR-17↑ → Activation of pathway TLR4/NF-κB↑ → proinflammatory cytokines↑ → proliferation↑, migration↑, and invasion of endometrial stromal cells (ESCs)↑, apoptosis↓/ Treatment research: levonorgestrel treatment in AM→ miR-17↓	Not investigated
Lin, 2020 [27]	Junctional zone (JZ) tissue (sample taken after hysterectomy), proliferative phase	RT-qPCR	1	None	Let-7a	Lin28B↑ → Let-7a↓→ proliferation of junctional zone smooth muscle cells (JZSMCs)↑	Not investigated
Huang N, 2021 [28]	Eutopic endometrial tissue (sample taken after hysterectomy), proliferative phase	RT-qPCR	1	None	miR-124-3p	miR-124-3p↓ → Neuropilin-1 (NRP1)↑ → enhanced migration of ESCs, Behavior of EMT markers (E-cadherin↓, N-cadherin↑, Vimentin↑, MMP-9↑)	Not investigated
Huang JH, 2021 [29]	JZ tissue (sample taken after hysterectomy)	RT-qPCR	1	None	Let-7a	let7a↓ → Hippo-YAP 1 pathway↑ (YAP1 and TAZ↑) → proliferation of junctional zone smooth muscle cells (JZSMCs)↑, apoptosis↓	Not investigated
Huang JH, 2021 [30]	JZ tissue (sample taken after hysterectomy)	RT-qPCR	1	None	Let-7a	17β-estradiol → Lin28B↑ → Let-7a↓ → proliferation of junctional zone smooth muscle cells (JZSMCs)↑	Not investigated
Wang, 2021 [31]	Eutopic and ectopic endometrial tissue (sample taken after hysterectomy), proliferative or secretory phase	RT-qPCR	1	None	miR-145 (eutopic/ectopic endometrial tissue)	circPVT1↑ → miR-145↓ → Talin1↑ → Proliferation and Invasion of adenomyotic epithelial and stromal cells↑	Not investigated
Wang, 2021 [32]	Eutopic and ectopic endometrial tissue (sample taken after hysterectomy), proliferative or secretory phase	RT-qPCR	1	None	miR-145-5p (eutopic/ectopic endometrial tissue)	miR-145-5p↓ → Talin1↑ → Wnt/β-catenin pathway activation↑ → EMT↑ → migration and invasion adenomyotic epithelial cells↑	Not investigated
Yu, 2021 [33]	Eutopic endometrial tissue (biopsy), Menstruation (Cycle day 3)	RT-qPCR	1	None	miR-2861	MIR22HG↓ → miR-2861↓ (due to hypermethylation) → STAT3↑ and MMP2↑ → adenomyotic endometrial cell proliferation↑	Not investigated
Zhang, 2021 [34]	Eutopic and ectopic endometrial tissues (samples taken after hysterectomy)	RT-qPCR	1	None	miR-30c-5p	miR-30c-5p↓ → MAPK1↑ → proliferation, migration, invasion of adenomyotic epithelial cells↑	Low levels of miR-30c-5p correlated with the severity of the illness, such as dysmenorrhea, longer disease duration, and heavier menstrual bleeding in patients.
Li, 2022 [35]	Eutopic endometrial tissues (samples taken after hysterectomy), proliferative phase	RT-qPCR	1	None	miR-141-3p	circ_0061140↑ → miR-141-3p↓ → *LIN28B*↑ → apoptosis↓, cell migration↑ and invasion↑, cell viability↑, proliferation of endometrial epithelial cells↑	Not investigated
Wang and Chen, 2022 [36]	Eutopic endometrial tissue (biopsy), proliferative phase	RT-qPCR	1	None	miR-183	miR-183↓ → MMP-9↑ → viability, migration, invasion of adenomyotic epithelial cells↑	Not investigated
Zhang, 2022 [37]	Endometrial stromal cells (ESCs) of ectopic endometrial tissue (samples taken after hysterectomy), proliferative phase	RT-qPCR	1	None	miR-218-5p	miR-218↓ → LASP1↑ → Vimentin↑ → epithelial–mesenchymal transition (EMT) activation↑	Not investigated
Xu, 2023 [38]	Eutopic endometrial tissue (samples taken after hysterectomy)	RT-qPCR	1	miR-191	None	lncRNA MIR503HG↓ → miR-191↑ → Wnt/β-catenin↑ → cell proliferation↑, migration↑, invasion↑, EMT↑	Not investigated
Guo, 2024 [39]	Eutopic endometrial tissue (samples taken after hysterectomy)	RT-qPCR	1	miR-124-3p	None	circ_0008959↓ → miR-124-3p↑ → SLC15A4↓ → SLC15A4 may play a critical role in cell proliferation and invasion through regulating related pathways	Clinical Evaluation: miR-124-3p was identified as an independent risk factor for AM in multivariable logistic regression (OR = 2.55; 95% CI: 1.14–7.73; *p* = 0.046). Diagnostic Evaluation: Elevated miR-124-3p levels are associated with an increased likelihood of AM. ROC analysis showed good diagnostic performance (AUC = 0.875). Combined biomarker model (RNAs + VAS score) markedly improved diagnostic accuracy (AUC up to 0.976).
Hu, 2024 [40]	Extracellular vesicles of eutopic endometrial tissue (diagnostic curettage or hysterectomy sample) and peripheral blood (serum)	RT-qPCR	3	miR-25-3p (endometrium and serum)	None	miR-25-3p↑ → M2 macrophage polarization → EMT↑; endometrial epithelial cells: PTEN↓, AKT activation↑ → Migration↑	Not investigated
Wang, 2024 [41]	Endometrial–myometrial interface (EMI) tissue (samples taken after hysterectomy), isolation of primary smooth muscle cells (SMCs) = EMI SMCs	RT-qPCR	1	None	miR-141-3p	miR-141-3p↓ → activation of Janus Kinase 2/Signal Transducer and Activator of Transcription 3 (JAK2/STAT3)↑ → enhanced proliferation, reduced apoptosis	Not investigated
Zhang, 2024 [42]	Ectopic and eutopic endometrial tissue (samples taken after hysterectomy), isolation of ectopic ESCs	miRNA fluorescence in situ hybridization	2	miR-145 (ectopic endometrial tissue and ESCs)	None	E2↑ → miR-145↑ → CITED)↓ → NF-κB/HIF1α↑ → IL-1β/IL-6/VEGF↑ → promotion of angiogenesis and inflammation	Not investigated
Zipponi, 2025 [11]	Endometrial biopsies (via Novak curette), isolation of exosomes from ESCs, proliferative phase	Screening: NGS; validation: RT-qPCR	Screening: 2632; validation: 38	miR-132-5p miR-99a-5p miR-451a	miR-337-5p miR-590-3p miR-29c-3p miR-144-3p miR-7-5p miR-431-3p miR-1275	Most common target genes: B-cell lymphoma 2-gene (*BCL2*), B-cell translocation gene 2 (*BTG*), BTG antiproliferation factor 2, Akt signaling, aminoethanethiol dioxygenase (ADO), insulin-like growth factor 1 receptor (IGF1R), Nucleus accumbens-associated 1 (NACC1), Pleckstrin homology domain-containing A3 (PLEKHA3), SUZ RNA-binding domain-containing 1 (SZRD1) and Tet methylcytosine dioxygenase (TET)	Not investigated
Qiu, 2025 [43]	Endometrial and serum samples, exosomes of serum and isolated endometrial mesenchymal stem cells of eutopic endometrial tissue	RT-qPCR	1	miR-4669 (endometrium, serum)	None	miR-4669↑ → DUSP6↓ → ERK/MAPK↑ → M2 macrophage polarization↑ → TGF-β1 secretion↑ → epithelial–mesenchymal transition (EMT)↑ → migration and invasion↑	Clinical Evaluation: In patients with AM, serum exosomal miR-4669 levels were significantly elevated and positively correlated with the PBAC score, VAS pain score, and uterine volume, suggesting a relationship between circulating miR-4669 levels and disease severity.
Shao, 2025 [44]	Exosomes from plasma, urine and eutopic endometrial tissue and ectopic lesions of AM	RT-qPCR	82	miR-92a-3p (endometrium, plasma, urine)	None	miR-92a-3p↑ → PTEN↓ → PI3K/AKT↑ → enhanced cell proliferation, migration, invasion, and angiogenesis	Clinical evaluation and diagnostic approach: MiR-92a-3p was consistently upregulated in plasma exosomes, exosomes from ectopic lesions, and urinary exosomes from patients with AM, and its expression level correlated positively with clinical severity parameters. Among these compartments, miR-92a-3p showed the highest diagnostic accuracy in urinary exosomes.
Zhou, 2025 [45]	Serum, proliferative phase (cycle day 5–14)	Screening: NGS; validation: RT-qPCR	Screening: 222; validation: 2	miR-101-3p miR-143-3p	None	Not specified	Diagnostic approach: This study introduces a serum 2-miRNA panel (miR101-3p and miR-143-3p) as a high-accuracy diagnostic tool for AM, capable of distinguishing AM from endometriosis, uterine fibroids, endometrial polyps, and healthy controls.
Kupec, 2025 [46]	Serum, urine	NGS	Screening: 4285; validation: 20	Not specified	Not specified	Not specified	Diagnostic approach: Most relevant and consistent miRNA in urine: miR-183-3p, miR-8077; in serum: miR-17, miR-3132, miR-5186, and miR-4446; and in both (urine and serum): miR-320d-2.

Abbreviations: Adenomyosis (AM), area under the curve (AUC), Dual-specificity phosphatase 6 (DUSP6), endometrial–myometrial interface (EMI), epithelial–mesenchymal transition (EMT), endometrial stromal cell (ESC), Extracellular signal-regulated kinase/mitogen-activated protein kinase (ERK/MAPK), interleukin (IL), Janus Kinase 2/Signal Transducer and Activator of Transcription 3 (JAK2/STAT3), LIM and SH3 domain protein (LASP1), Let-7 (miRNA Lethal-7), Lin-28 RNA Binding Posttranscriptional Regulator B (LIN28B), Matrix Metalloproteinase-9 (MMP-9), next-generation sequencing (NGS), junctional zone (JZ), Nuclear factor kappa-light-chain-enhancer of activated B cells/Hypoxia-inducible factor 1-alpha (NF-κB/HIF1α), Phosphatase and tensin homolog (PTEN), Phosphoinositide 3-kinase/Protein Kinase B (PI3K/AKT), Pictorial Bleeding Assessment Chart (PBAC), Receiver Operating Characteristic (ROC), reverse transcription–quantitative polymerase chain reaction (RT-qPCR), smooth muscle cells (SMCs), Solute Carrier Family 15 Member 4 (SLC15A4), Transforming growth factor beta 1 (TGF-β1), Vascular Endothelial Growth Factor (VEGF), Visual Analog Scale (VAS), Wingless-type MMTV integration site family β-catenin pathway (Wnt/β). Symbols: → regulation; ↑ upregulated/increased; ↓ downregulated/decreased.

**Table 2:** Thirty-nine significant dysregulated MiRNAs in AM in different tissue types.

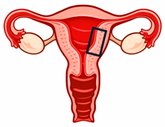		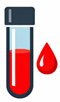	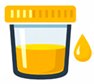
Endometrial Tissue		Plasma/Serum	Urine
miRNAs ↑	miRNAs ↓	miRNAs ↑	miRNAs ↑
miR-9-1 **miR-17** *miR-25-3p* *miR-92a-3p* miR-99a-5p **miR-124-3p** miR-132-5p miR-139 **miR-143** **miR-145** miR-149 **miR-191** miR-197 miR-326 miR-339 miR-451a miR-466 miR-513a *miR-4669*	**Let-7a** miR-7-5p **miR-10b** **miR-21** miR-29c-3p **miR-30c-5p** miR-92b-5p **miR-124-3p** **miR-141-3p** miR-144-3p **miR-145** miR-183 miR-200c miR-218-5p miR-337-5p miR-371b-5p miR-431-3p miR-590-3p miR-1275 miR-2861	*miR-25-3p* *miR-92a-3p* miR-101-3p miR-143-3p *miR-4669*	*miR-92a-3p*

Legend: Bold: multiple mentions in more than one study; italics: multiple mentions in different tissues; underline: multiple mentions of up- and downregulation; ↑: upregulated; ↓: downregulated). Pictures were generated using ChatGPT-5.5 (OpenAI) based on a prompt written by the authors. The authors reviewed and edited the generated images and take full responsibility for their content.

## Data Availability

No new data were created or analyzed in this study.

## Supplementary Materials

The following supporting information can be downloaded at: https://www.mdpi.com/article/10.3390/biomedicines14081764/s1, File S1: Complete database-specific search strategies for all databases, File S2: Table S1: Additional information regarding study characteristics; Table S2: Newcastle–Ottawa quality assessment form for cohort studies; Table S3: PRISMA Checklist 2020.

## Abbreviations

The following abbreviations are used in this manuscript:

AM	Adenomyosis
AUC	Area under the curve
ART	Assisted reproductive technology
DUSP6	Dual-specificity phosphatase 6
EMI	Endometrial–myometrial interface
EMT	Epithelial–mesenchymal transition
ESC	Endometrial stromal cell
ERK/MAPK	Extracellular signal-regulated kinase/mitogen-activated protein kinase
JAK2/STAT3	Janus Kinase 2/Signal Transducer and Activator of Transcription 3
JZ	Junctional zone
LASP1	LIM and SH3 domain protein
Let7	miRNA Lethal-7
LIN28B	Lin-28 RNA Binding Posttranscriptional Regulator B
MMP-9	Matrix Metalloproteinase-9
ML	Machine learning
miRNA/miR	MicroRNA
MRI	Magnetic resonance imaging
NGS	Next-generation sequencing
NF-κB/HIF1α	Nuclear factor kappa-light-chain-enhancer of activated B cells/Hypoxia-inducible factor 1-alpha
PBAC	Pictorial Bleeding Assessment Chart
PI3K/AKT	Phosphoinositide 3-kinase/Protein Kinase B Pathway
PTEN	Phosphatase and tensin homolog
ROC	Receiver Operating Characteristic
RT-qPCR	Reverse transcription–quantitative polymerase chain reaction
SMC	Smooth muscle cell
TGF-β1	Transforming growth factor beta 1
TIAR	Tissue injury and repair
TVUS	Transvaginal ultrasound
VEGF	Vascular Endothelial Growth Factor
VAS	Visual Analog Scale
Wnt/β	Wingless-type MMTV integration site family β-catenin pathway
