# Supplementary material for: MicroRNAs as Biomarkers for Adenomyosis: A Systematic Review
Source: Biomedicines. 2026 Aug 5;14(8):1764. doi: 10.3390/biomedicines14081764 (PMC13509202; doi:10.3390/biomedicines14081764)
Supplement: Supplementary file 1 [file biomedicines-14-01764-s001.zip › biomedicines-4412201-File S1.pdf]

File S1

Detailed search strategies

- OVERVIEW -

Search date	Database searched	Platform	Database coverage (inception)	Records retrieved	Records after deduplication (Deduklick)
Apr 29, 2025	Medline	Ovid (Wolters Kluwer)	1946 to Apr 28, 2025	410	406
Apr 29, 2025	Embase	Ovid (Wolters Kluwer)	1974 – Apr 28, 2025	966	627
Apr 29, 2025	Scopus	Elsevier	2004 - present	925	249
Apr 29, 2025	Cochrane Central Register of Controlled Trials (CENTRAL)	Wiley	1995 - present	27	14
Apr 29, 2025	Cochrane Database of Systematic Reviews (CDSR)	Wiley	1996- present	0	0
			**Total**	2328	**1296**

- DATABASE SEARCHES –

Medline/Ovid

Search date: April 29, 2025

Ovid MEDLINE(R) ALL <1946 to April 28, 2025>		
1	Adenomyosis/	1568
2	(Adenomyos* or endometrial invasion* or endometrial infiltrat* or (ectopic endometrium adj2 myometrium) or internal endometrios* or (hypertroph* adj3 myometri*) or endometrial adenoma* or (endometriosis adj2 uterin*) or stromal endometrios*).ti,ab,kf.	5044
3	1 or 2	5094
4	exp MicroRNAs/ or biomarkers/ or genetic markers/	575073
5	(mirna* or microrna* or mi-rna* or micro-rna* or non-coding rna* or noncoding rna* or small temporal rna* or biomarker* or biologic* marker* or bio-marker* or small rna* or Mir* or mir-21* or mir-155* or mir-200* or mir-451* or mir-223* or mir-126* or mir-146* or hsa-mir*).ti,ab,kf.	834092
6	4 or 5	1123411
7	Proto-Oncogene Proteins c-bcl-2/ or exp Cadherins/ or Cell Survival/ or Apoptosis/ or exp Gene Expression/ or exp Apoptosis Regulatory Proteins/ or exp Apoptosis Regulatory Proteins/ or Exosomes/ or bcl-2-Associated X Protein/ or exp Caspases/	1110531

8	(Paracine signal* or Extracellular vesicle* or gene express* or exosom* or EV-mediated or pten or zeb1 or pik3ca or mmp-9 or incrn* or Bcl-2 or Bax or E-cadherin* or Aktphosphorylation or cell viabilit* or apoptos* or antiapoptos*).ti,ab,kf.	1250826
9	7 or 8	1846500
10	3 and (6 or 9)	485
11	(exp animals/ or exp animal experimentation/ or exp models, animal/ or exp plants/ or exp fungi/) not humans/	5797121
12	10 not 11	453
13	limit 12 to yr="2000 -Current"	435
14	limit 13 to (english or french or german or spanish)	410
Link to search		

Embase/Ovid

Search date: April 29, 2025

Embase <1974 to 2025 April 28>		
1	(Adenomyos* or endometrial invasion* or endometrial infiltrat* or (ectopic endometrium adj2 myometrium) or internal endometrios* or (hypertroph* adj3 myometri*) or endometrial adenoma* or (endometriosis adj2 uterin*) or stromal endometrios*).ti,ab,kf.	7558
2	exp adenomyosis/	8067
3	1 or 2	10073
4	exp microRNA/ or exp biological marker/	769831
5	(mirna* or microrna* or mi-rna* or micro-rna* or non-coding rna* or noncoding rna* or small temporal rna* or biomarker* or biologic* marker* or bio-marker* or small rna* or Mir* or mir-21* or mir-155* or mir-200* or mir-451* or mir-223* or mir-126* or mir-146* or hsa-mir*).ti,ab,kf.	1138016
6	4 or 5	1300755
7	(Paracine signal* or Extracellular vesicle* or gene express* or exosom* or EV-mediated or pten or zeb1 or pik3ca or mmp-9 or incrn* or Bcl-2 or Bax or E-cadherin* or Aktphosphorylation or cell viabilit* or apoptos* or antiapoptos*).ti,ab,kf.	1643375
8	exp protein bcl 2/ or exp uvomorulin/ or apoptosis/ or cell viability/ or exp gene expression/ or exp exosome/ or apoptosis regulatory protein/ or caspase/ or caspase 3/	3082650
9	7 or 8	3404086
10	(exp animal/ or exp invertebrate/ or nonhuman/ or animal experiment/ or animal tissue/ or animal model/ or exp plant/ or exp fungus/) not (exp human/ or human tissue/)	8250984
11	(3 and 6) not 10	379
12	(3 and (6 or 9)) not 10	1049
13	limit 12 to yr="2000 -Current"	1026
14	limit 13 to (english or french or german or spanish)	966
Link to search		

Scopus/Elsevier

Search date: April 29, 2025

(TITLE-ABS-KEY (adenomyos* OR "endometrial invasion*" OR "endometrial infiltrat*" OR ("ectopic endometrium" W/2 myometrium) OR "internal endometrios*" OR (hypertroph* W/3 myometri*) OR "endometrial adenoma*" OR (endometriosis W/2 uterin*) OR "stromal endometrios*")) AND ((TITLE-ABS-KEY (("Paracine signal*" OR "Extracellular vesicle*" OR "gene express*" OR exosom* OR ev-mediated OR pten OR zeb1 OR pik3ca OR mmp-9 OR incrn* OR bcl-2 OR bax OR e-cadherin* OR aktphosphorylation OR "cell viabilit*" OR apoptos* OR antiapoptos*))) OR (TITLE-ABS-KEY ((mirna* OR microrna* OR mi-rna* OR micro-rna* OR "non-coding rna*" OR "noncoding rna*" OR "small temporal rna*" OR biomarker* OR "biologic* marker*" OR bio-marker* OR "small rna*" OR mir* OR mir-21* OR mir-155* OR mir-200* OR mir-451* OR mir-223* OR mir-126* OR mir-146* OR hsa-mir*)))) AND PUBYEAR > 1999 AND PUBYEAR < 2026 AND (LIMIT-TO (LANGUAGE , "English") OR LIMIT-TO (LANGUAGE , "German") OR LIMIT-TO (LANGUAGE , "French") OR LIMIT-TO (LANGUAGE , "Spanish")
Results: 925

Cochrane Database/Wiley

Search date: April 29, 2025

#1	[mh ^Adenomyosis]	74
#2	(Adenomyos* OR ("endometrial" NEXT invasion*) OR ("endometrial" NEXT infiltrat*) OR ("ectopic endometrium" NEAR/2 myometrium) OR ("internal" NEXT endometrios*) OR (hypertroph* NEAR/3 myometri*) OR ("endometrial" NEXT adenoma*) OR (endometriosis NEAR/2 uterin*) OR ("stromal" NEXT endometrios*)):ti,ab,kw	539
#3	#1 OR #2	539
#4	[mh MicroRNAs] OR [mh ^biomarkers] OR [mh ^"genetic markers"]	21698
#5	(mirna* OR microrna* OR mi-rna* OR micro-rna* OR ("non-coding" NEXT rna*) OR ("noncoding" NEXT rna*) OR ("small temporal" NEXT rna*) OR biomarker* OR (biologic* NEXT marker*) OR bio-marker* OR ("small" NEXT rna*) OR Mir* OR mir-21* OR mir-155* OR mir-200* OR mir-451* OR mir-223* OR mir-126* OR mir-146* OR hsa-mir*):ti,ab,kw	71170
#6	#4 OR #5	71452
#7	[mh ^"Proto-Oncogene Proteins c-bcl-2"] OR [mh Cadherins] OR [mh ^"Cell Survival"] OR [mh ^Apoptosis] OR [mh "Gene Expression"] OR [mh "Apoptosis Regulatory Proteins"] OR [mh "Apoptosis Regulatory Proteins"] OR [mh ^Exosomes] OR [mh ^"bcl-2-Associated X Protein"] OR [mh Caspases]	3434
#8	(("Paracine" NEXT signal*) OR ("Extracellular" NEXT vesicle*) OR ("gene" NEXT express*) OR exosom* OR EV-mediated OR pten OR zeb1 OR pik3ca OR mmp-9 OR incrn* OR Bcl-2 OR Bax OR E-cadherin* OR Aktphosphorylation OR ("cell" NEXT viabilit*) OR apoptos* OR antiapoptos*):ti,ab,kw	29913
#9	#7 OR #8	30640
#10	#6 OR #9	94523
#11	#3 AND #10 with Cochrane Library publication date Between Jan 2000 and May 2025, in Cochrane Reviews, Trials	27
Link to search		
