# Supplementary material for: MicroRNAs as Biomarkers for Adenomyosis: A Systematic Review
Source: Biomedicines. 2026 Aug 5;14(8):1764. doi: 10.3390/biomedicines14081764 (PMC13509202; doi:10.3390/biomedicines14081764)
Supplement: Supplementary file 1 [file biomedicines-14-01764-s001.zip › biomedicines-4412201-File S2.pdf]

File S2

Table S1. Additional information regarding study characteristics

Author, Year	Sample Size (Case/ Control)	Diagnostic method for adenomyosis	Previous hormonal treatment/ exclusion criteria (all patients)	Control group characteristics
Guo, 2015 [19]	22/ 13	Histopathology after hysterectomy	Not specified	Premenopausal women with hysterectomy for subserosal leiomyoma.
Herndon, 2016 [20]	3/ 5	Histopathology after hysterectomy	No hormone therapy ≥3 months/ no endometriosis, no fibroids, no pregnancy	Premenopausal women with normo-ovulatory cycles undergoing endometrial biopsy for non-malignant surgical indication
Hu, 2017 [21]	45/ 32	Clinically and pathologically	No hormone therapy ≥3 months	Premenopausal women undergoing hysteroscopy for abnormal uterine mediastinum, no other disease
Yan, 2019 [22]	21/ 36	Transvaginal ultrasound (≥2 of 5 sonographic criteria: indistinct junctional zone, asymmetry, striations, cysts/fibrosis, heterogeneous myometrium) + elevated CA-125 (≥35 U/mL)	No hormone therapy ≥3 months/ no regular cycles, no PCOS, hydrosalpinx, endometrial hyperplasia, no endometrial polyps	Fertile women with ≥1 normal pregnancy and/or delivery
Borisov, 2020 [23]	33/ 30	Complaints, physical examination, Transvaginal ultrasound (TVUS) -2 US specialists using MUSA criteria, and MRI	No medications ≥4 months/ no autoimmune or metabolic disease, no pelvic inflammatory disease, no myoma, no fibroids, no dysfunctional uterine bleeding.	Healthy women
Liang, 2020 [24]	71/ 54	Clinically and pathologically	Not specified	Women undergoing hysteroscopic uterine septum resection, no history of hormone therapy, no underlying chronic diseases, and no mental diseases
Lin, 2020 [25]	30/ 30	Histopathology after hysterectomy: clinical histopathological classification as diffuse adenomyosis, including JZ thickening and outer	No hormone therapy ≥3 months/ no fibroids, no endometrial polyps, no endometrial cancer, no irregular menstruation	Premenopausal women with early-stage cervical cancer or uterine prolapse

		myometrium extensive disease with endometrial glands and stroma scattered throughout the uterine musculature		
Huang N, 2021 [26]	20/ 20	Histopathology after hysterectomy	No hormone therapy ≥3 months	Women undergoing hysterectomy for benign conditions of uterine leiomyoma
Huang JH, 2021 [27]	25/ 27	Histopathology after hysterectomy	No hormone therapy ≥3 months/ no fibroid, no endometriosis or endometrial pathology or malignancy	Premenopausal women with early-stage cervical or ovarian cancer
Huang JH, 2021 [28]	20/ 20	Histopathology after hysterectomy	No hormone therapy ≥3 months/ no irregular menstruation, no endometrial polyps, no fibroids, no pelvic inflammation, no endometrial and myometrial cancer, and no preoperative radiotherapy and chemotherapy	Premenopausal women with early-stage cervical cancer; early-stage ovarian cancer or uterine prolapse
Wang, 2021 [29]	45/ 40	Histopathology after hysterectomy	No hormone therapy ≥3 months/ no irregular menstrual cycles, no endometriosis, no endometrial pathology or malignancy	Premenopausal women with early-stage ovarian cancer or cervical cancer
Wang, 2021 [30]	42/ 38	Histopathology after hysterectomy	No hormone therapy ≥3 months/ no irregular menstrual, no endometriosis, no endometrial carcinoma, no polyps or other pathology	Premenopausal women, no AM
Yu, 2021 [31]	45/ 45	Transvaginal sonography (2/5 Musa criteria)	No initiated therapy for AM/ no endometrial disease, no initiated therapy for AM	Control patients received systemic physiological exam at aforementioned hospital, and all physiological function indicators of all controls were within the normal range
Zhang, 2021 [32]	23/ 20	Histopathology after hysterectomy	Not specified	Premenopausal women who underwent a hysterectomy with benign gynecological diseases, such as uterine prolapse and subserosal leiomyoma

Li, 2022 [33]	27/ 15	Histopathology after hysterectomy	No hormone therapy ≥3 months/ / no irregular menses	Premenopausal women who underwent hysterectomy for uterine fibroids, no history of hormone therapy, no underlying chronic diseases, and no mental diseases
Wang and Chen, 2022 [34]	30/ 30	Not specified	No hormone therapy	Women with normal endometrium
Zhang, 2022 [35]	3/ 1 (cell line)	Histopathology after hysterectomy (Endometrial stroma and glands invade the myometrium more than 2 mm)	No hormone therapy ≥3 months	The control ESCs (ThESC/Group1) were obtained from ATCC (CRL-4003, a hTERT-immortalized endometrium stromal cell line from an adult female with non-malignant myomas)
Xu, 2023 [36]	30 /30	Clinically and pathologically	No prior hormone therapy/ no chronic disease	Patients who underwent hysterectomy with cervical lesions or uterine fibroids
Guo, 2024 [37]	48/ 39	Histopathology after hysterectomy (two pathologists)	No hormone therapy ≥3 months/ no endometrial disease, no pelvic inflammatory disease, no chronic diseases	Premenopausal women with hysterectomy for cervical intraepithelial neoplasia (CIN III)
Hu, 2024 [38]	11/ 10	Histopathology after hysterectomy or resection of adenomyosis lesions and diagnostic curettage	No hormone therapy ≥3 months/ no endometriosis	Premenopausal women with tubal obstruction
Wang, 2024 [39]	25/ 20	Histopathology after hysterectomy	No hormone therapy ≥3 months/ no irregular menstrual cycles, no endometriosis, no pelvic inflammation, no malignancy of endometrium/myometrium, no prior chemo/radiotherapy	Premenopausal patients with who underwent hysterectomy for grade III cervical intraepithelial neoplasia, early-stage cervical/ovarian cancer)
Zhang, 2024 [40]	28 /14	Transvaginal ultrasound, MRI or CT; presentation of significant symptoms, such as dysmenorrhoea and menstrual irregularity, confirmation by histopathology after hysterectomy	No hormone therapy ≥3 months/ no irregular menstrual cycle, pregnancy, lactation, no tumors of reproductive system, no endometrial lesions, no severe liver or kidney dysfunction, no haematologic disease, no endocrine disorders	Premenopausal women without adenomyosis undergoing hysterectomy
Zipponi, 2025 [11]	15/ 15	MRI or transvaginal sonography	No hormone therapy ≥2 months/ no irregular menstrual cycle	Premenopausal women with no adenomyosis, no endometriosis,

				and no other endometrial pathology
Qiu, 2025 [41]	3 (endometrial tissue), 36 (serum)/ 3 (endometrial tissue), 36 (serum)	Histopathology after hysterectomy; no specification for patients of the serum sample	No hormone therapy ≥3 months	Patients with ovarian tumors
Shao, 2025 [42]	45 (plasma), 40 (exosomes), 41 (urine)/ 21 (plasma), 19 (exosomes), 16 (urine)	Histopathology after hysterectomy	No hormone therapy ≥3 months/ no smoking, and no history of other inflammatory diseases requiring surgical treatment. no co-existing malignant tumors, no pregnancy, no endometriosis	Premenopausal women who had undergone hysterectomy for uterine leiomyomas
Zhou, 2025 [43]	30 (screening); 45 (validation), 30 (blinded validation)/ 30 (screening); 45 (validation), 30 (blinded validation)	Histology	No hormone therapy ≥6 months/ no other gynecological comorbidities, no pregnancy, no serious organic or functional disorders	Healthy women undergoing routine physical examinations at the same institutions and showing normal results, and 3 other groups with patients with endometriosis, uterine fibroids, and endometrial polyps
Kupec, 2025 [44]	7/ 34 (endometriosis), 18 (no endometriosis)	Preoperative transvaginal ultrasound (TVUS, MUSA criteria) and intraoperative assessment	Not specified	Patients who underwent surgery for suspected endometriosis but in whom endometriosis and adenomyosis were excluded intraoperatively, and patients with endometriosis

Table S2: Newcastle-Ottawa quality assessment form for cohort studies

First author, Year of publication	Is the case definition adequate:	Representativeness of the cases:	Selection of controls:	Definition of controls:	Comparability of cases and controls based on the design or analysis controlled for confounders:	Ascertainment of exposure:	Same method of ascertainment for cases and controls:	Non-response rate:	Total	Quality Assessment
	a) Yes, with independent validation (one star) b) Yes, e.g., record linkage or based on self-report c) No description	a) Consecutive or obviously representative series of cases (one star) b) Potential for selection biases or not stated	a) Community controls (one star) b) Hospital controls c) No description	a) No history of disease (endpoint) (one star) b) No description of source	a)The study controls for age (one star) b) Study controls for other factors (list)(one star) c) Cohorts are not comparable on the basis of the design or analysis controlled for confounders	a) Secure record (one star) b) Structured interview where blind to case/control status (one star) c) Interview not blinded to case/control status d) Written self-report or medical record only e) No description	a) Yes (one star) b) No	a) Same rate for both groups (one star) b) Non-respondents described c) Rate different between cases and controls with no description		
Guo, 2015 (1)	★	★	*0*	★	★	★	★	*0*	*6*	*Fair*
Herndon, 2016 (2)	★	★	*0*	★	★	★	★	*0*	*6*	*Fair*
Hu, 2017 (3)	*0*	★	*0*	★	*0*	★	★	*0*	*5*	*Poor*
Yan, 2019 (4)	*0*	★	*0*	★	★	★	★	*0*	*5*	*Fair*

Borisov, 2020 (5)	★	★	*0*	★	★	★	★	*0*	*6*	*Fair*
Liang, 2020 (6)	★	★	*0*	★	*0*	★	★	*0*	*4*	*Poor*
Lin, 2020 (7)	★	★	*0*	★	★	★	★	*0*	*6*	*Fair*
Huang N, 2021 (8)	★	★	*0*	★	★	★	★	*0*	*6*	*Fair*
Huang JH, 2021 (9)	★	★	*0*	★	★	★	★	*0*	*6*	*Fair*
Huang JH, 2021 (10)	★	★	*0*	★	★	★	★	*0*	*6*	*Fair*
Wang, 2021 (11)	★	★	*0*	★	★	★	★	*0*	*6*	*Fair*
Wang, 2021 (12)	★	★	*0*	★	★	★	★	*0*	*6*	*Fair*
Yu, 2021 (13)	*0*	★	★	★	★	★	★	*0*	*6*	*Fair*
Zhang, 2021 (14)	★	★	*0*	★	★	★	★	*0*	*6*	*Fair*
Li, 2022 (15)	★	★	*0*	★	★	★	★	*0*	*6*	*Fair*
Wang and Chen, 2022 (16)	*0*	*0*	*0*	★	★	★	★	*0*	*4*	*Fair*
Zhang, 2022 (17)	★	*0*	*0*	★	*0*	★	★	*0*	*4*	*Poor*
Xu, 2023 (18)	★	★	*0*	★	★	★	★	*0*	*6*	*Fair*
Guo, 2024 (19)	★	★	*0*	★	★★	★	★	*0*	*7*	*Good*
Hu, 2024 (20)	★	★	*0*	★	*0*	★	★	*0*	*5*	*Poor*
Wang, 2024 (21)	★	★	*0*	★	★	★	★	*0*	*6*	*Fair*
Zhang, 2024 (22)	★	★	*0*	★	★	★	★	*0*	*6*	*Fair*
Zipponi, 2025	*0*	★	*0*	★	★	★	★	*0*	*5*	*Fair*

(23)										
Qiu, 2025 (24)	★	★	*0*	★	★	★	★	*0*	*6*	*Fair*
Shao, 2025 (25)	★	★	*0*	★	★	★	★	*0*	*6*	*Fair*
Zhou, 2025 (26)	★	★	*0*	★	*0*	*0*	★	*0*	*4*	*Poor*
Kupec, 2025 (27)	★	*0*	*0*	★	★	★	★	*0*	*5*	*Fair*

Note: A study can be awarded a maximum of one star for each numbered item within the Selection and Exposure categories. A maximum of two stars can be given for Comparability.

Table S3 Prisma Checklist 2020

Section and Topic	Item #	Checklist item	Location where item is reported Page
**TITLE**			
Title	1	Identify the report as a systematic review.	1
**ABSTRACT**			
Abstract	2	See the PRISMA 2020 for Abstracts checklist.	1
**INTRODUCTION**			
Rationale	3	Describe the rationale for the review in the context of existing knowledge.	2
Objectives	4	Provide an explicit statement of the objective(s) or question(s) the review addresses.	2
**METHODS**			
Eligibility criteria	5	Specify the inclusion and exclusion criteria for the review and how studies were grouped for the syntheses.	3
Information sources	6	Specify all databases, registers, websites, organizations, reference lists and other sources searched or consulted to identify studies. Specify the date when each source was last searched or consulted.	3
Search strategy	7	Present the full search strategies for all databases, registers and websites, including any filters and limits used.	3
Selection process	8	Specify the methods used to decide whether a study met the inclusion criteria of the review, including how many reviewers screened each record and each report retrieved, whether they worked independently, and if applicable, details of automation tools used in the process.	4

Section and Topic	Item #	Checklist item	Location where item is reported Page
Data collection process	9	Specify the methods used to collect data from reports, including how many reviewers collected data from each report, whether they worked independently, any processes for obtaining or confirming data from study investigators, and if applicable, details of automation tools used in the process.	5
Data items	10a	List and define all outcomes for which data were sought. Specify whether all results that were compatible with each outcome domain in each study were sought (e.g. for all measures, time points, analyses), and if not, the methods used to decide which results to collect.	5
	10b	List and define all other variables for which data were sought (e.g. participant and intervention characteristics, funding sources). Describe any assumptions made about any missing or unclear information.	5
Study risk of bias assessment	11	Specify the methods used to assess risk of bias in the included studies, including details of the tool(s) used, how many reviewers assessed each study and whether they worked independently, and if applicable, details of automation tools used in the process.	5
Effect measures	12	Specify for each outcome the effect measure(s) (e.g. risk ratio, mean difference) used in the synthesis or presentation of results.	5
Synthesis methods	13a	Describe the processes used to decide which studies were eligible for each synthesis (e.g. tabulating the study intervention characteristics and comparing against the planned groups for each synthesis (item #5)).	5
	13b	Describe any methods required to prepare the data for presentation or synthesis, such as handling of missing summary statistics, or data conversions.	5
	13c	Describe any methods used to tabulate or visually display results of individual studies and syntheses.	5
	13d	Describe any methods used to synthesize results and provide a rationale for the choice(s). If meta-analysis was performed, describe the model(s), method(s) to identify the presence and extent of statistical heterogeneity, and software package(s) used.	5
	13e	Describe any methods used to explore possible causes of heterogeneity among study results (e.g. subgroup analysis, meta-regression).	5
	13f	Describe any sensitivity analyses conducted to assess robustness of the synthesized results.	5
Reporting bias assessment	14	Describe any methods used to assess risk of bias due to missing results in a synthesis (arising from reporting biases).	5
Certainty assessment	15	Describe any methods used to assess certainty (or confidence) in the body of evidence for an outcome.	5
**RESULTS**			

Section and Topic	Item #	Checklist item	Location where item is reported Page
Study selection	16a	Describe the results of the search and selection process, from the number of records identified in the search to the number of studies included in the review, ideally using a flow diagram.	6
	16b	Cite studies that might appear to meet the inclusion criteria, but which were excluded, and explain why they were excluded.	
Study characteristics	17	Cite each included study and present its characteristics.	7-13
Risk of bias in studies	18	Present assessments of risk of bias for each included study.	16
Results of individual studies	19	For all outcomes, present, for each study: (a) summary statistics for each group (where appropriate) and (b) an effect estimate and its precision (e.g. confidence/credible interval), ideally using structured tables or plots.	7-13
Results of syntheses	20a	For each synthesis, briefly summarise the characteristics and risk of bias among contributing studies.	14-16
	20b	Present results of all statistical syntheses conducted. If meta-analysis was done, present for each the summary estimate and its precision (e.g. confidence/credible interval) and measures of statistical heterogeneity. If comparing groups, describe the direction of the effect.	14-16
	20c	Present results of all investigations of possible causes of heterogeneity among study results.	14-16
	20d	Present results of all sensitivity analyses conducted to assess the robustness of the synthesized results.	14-16
Reporting biases	21	Present assessments of risk of bias due to missing results (arising from reporting biases) for each synthesis assessed.	14-16
Certainty of evidence	22	Present assessments of certainty (or confidence) in the body of evidence for each outcome assessed.	
**DISCUSSION**			
Discussion	23a	Provide a general interpretation of the results in the context of other evidence.	16-18
	23b	Discuss any limitations of the evidence included in the review.	18
	23c	Discuss any limitations of the review processes used.	18
	23d	Discuss implications of the results for practice, policy, and future research.	18
**OTHER INFORMATION**			

Section and Topic	Item #	Checklist item	Location where item is reported Page
Registration and protocol	24a	Provide registration information for the review, including register name and registration number, or state that the review was not registered.	3
	24b	Indicate where the review protocol can be accessed, or state that a protocol was not prepared.	3
	24c	Describe and explain any amendments to information provided at registration or in the protocol.	3
Support	25	Describe sources of financial or non-financial support for the review, and the role of the funders or sponsors in the review.	18
Competing interests	26	Declare any competing interests of review authors.	18
Availability of data, code and other materials	27	Report which of the following are publicly available and where they can be found: template data collection forms; data extracted from included studies; data used for all analyses; analytic code; any other materials used in the review.	18

From: Page MJ, McKenzie JE, Bossuyt PM, Boutron I, Hoffmann TC, Mulrow CD, et al. The PRISMA 2020 statement: an updated guideline for reporting systematic reviews. BMJ 2021;372:n71. doi: 10.1136/bmj.n71. This work is licensed under CC BY 4.0. To view a copy of this license, visit https://creativecommons.org/licenses/by/4.0/
